# Health promotion at the crossroads: putting civil society in the driver’s seat

**DOI:** 10.1093/heapro/daag048

**Published:** 2026-04-09

**Authors:** Veronica Shiroya, Thelma Bost, Bernard Kadasia, Gabriella Sozanski

**Affiliations:** Alliance for Health Promotion, Avenue de Sécheron 15, Geneva CH-1202, Switzerland; Centre for Preventive Medicine and Digital Health, Medical Faculty Mannheim, Heidelberg University, Röntgenstraße 7, Mannheim 68167, Germany; Heidelberg Institute of Global Health, Heidelberg University Hospital, Im Neuenheimer Feld 672, Heidelberg 69120, Germany; Alliance for Health Promotion, Avenue de Sécheron 15, Geneva CH-1202, Switzerland; Alliance for Health Promotion, Avenue de Sécheron 15, Geneva CH-1202, Switzerland; Health Promotion Alliance of Kenya, Kitale 30200, Kenya; Alliance for Health Promotion, Avenue de Sécheron 15, Geneva CH-1202, Switzerland

Four decades after its adoption, the Ottawa Charter for Health Promotion (hereafter: Charter) still envisions a world that we have yet to build, one where people and communities increase control over their health and where policy, place, and participation are as important as clinical care (Lunnay *et al*. 2025). We are living in an era of ecological, digital, political, and social shocks that test the resilience of every system and community (Thomas *et al*. 2026). To meet these challenges, we must fulfil the Charter’s core vision and empower civil society as a leading partner by moving it to the centre of modern health promotion.

Ottawa’s five action areas (build healthy public policy, create supportive environments, strengthen community action, develop personal skills, and reorient health services) were designed to promote power within communities. It also insisted that community organizations are essential to public health (WHO 1986). However, early implementation reflected state-centric governance and a health sector more comfortable delivering services than sharing power (Thomas *et al*. 2026). This tension between a community-first charter and government-first practice has shaped the field ever since.

A notable outcome of the Charter was the concept of healthy cities. By embedding health in urban planning, transportation, housing, and governance, the approach demonstrated that cross-sector action and citizen participation can influence health determinants (Amri *et al*. 2022). However, its success revealed that inspiring municipal cases are not the same as a global architecture for civil society participation.

In 1997, the Jakarta Declaration openly called for ‘new players for a new era’. Jakarta’s priorities (promote social responsibility, increase investments, consolidate partnerships, empower communities and individuals, and secure infrastructure) explicitly widened the reach beyond governments (WHO 1997). In response, a civil society coalition, the NGO Advisory Group on Health Promotion (renamed the Alliance for Health Promotion (A4HP) in 2008), was established to bridge international declarations and local realities (Sozanski *et al*. 2020).

While the Charter provides a compass, the movement needs a vehicle. Civil society organizations are well placed to translate its principles into concrete action by mobilizing communities and creating spaces for participation. In 2015, A4HP launched Health Promotion Day ‘Walk for Health and Happiness’, bringing together delegates and local associations in a public forum. By creating direct interaction between policy-makers and communities, the initiative moved health promotion from declaration to lived experience. A4HP has also institutionalized youth involvement in governance by bringing onto its board the International Federation of Medical Students Associations and the International Pharmaceutical Students Federation, turning youth engagement into decision-making power (Sozanski *et al*. 2020). These examples are practical answers to Ottawa’s implicit question of who will do the enabling.

Translation happens on the ground. Consensus-building avenues like regional workshops connect global strategies with local realities. Examples include annual workshops hosted by A4HP in Kitale, Kenya, and Bangalore, India. These foster community engagement, help set local health priorities, and provide a forum for review and accountability (AddictLab 2023). This approach moves beyond simple messaging to train local actors, expand access to essential care, and strengthen health literacy.

The Shanghai Declaration (2016) placed health promotion at the heart of the 2030 Agenda for Sustainable Development by emphasizing good governance, healthy cities, and health literacy as the connection between determinants and outcomes (WHO 2016). In Shanghai, civil society efforts were publicly recognized. A4HP’s ‘Harnessing Civic Engagement’ poster ranked second worldwide in response to the PEI Declaration and Call for Action, adopted at the 6th Global Forum on Health Promotion (PAHO 2017).

Five years later, the Geneva Charter for Well-being reframed the goal as well-being societies, identifying five coordinated action areas (value and protect the planet, design an equitable economy, develop public policy for the common good, achieve universal health coverage, address digital transformation) which align closely with the foundation of the Charter (WHO 2022). The Geneva Charter’s promise will remain abstract unless we resource the networks that can translate it into daily governance and community action. There is a strain in the global health architecture, as institutions confront overlapping crises and financial uncertainty, prompting debates about structural reform. This moment calls for shifting how civil society is positioned within global health governance, from observers to recognized institutional actors with defined roles (Musolino *et al*. 2024). Regional institutions, including the European Union and the African Union, are increasingly influential for health promotion governance as they offer opportunities to align policy, financing, and civil society participation closer to communities.

While platforms such as the WHO’s Global Health Promotion Hub (GHPH) offer valuable connective infrastructure, the role of civil society in health promotion governance cannot be confined to any single institutional mechanism (Musolino *et al*. 2024). The A4HP’s annual Global Forum, organized through collaboration with a variety of partners, demonstrates how governance processes can also be driven through independent civil society–led spaces (Sozanski *et al*. 2020). In addition, associations such as the Digital Transformations for Health Lab (DTH-Lab) are advancing work on youth engagement and digital determinants of health, aligning with the Geneva Charter’s call to confront digital harms while leveraging digital public goods (Holly *et al*. 2025). Coordinated action between civil society and scientific associations will be essential to ensure that health promotion practice remains evidence-based and responsive to emerging risks.

Two persistent barriers keep civil society from delivering at scale. The first is financing. We ask community organizations to do health promotion without the core funding that turns projects into institutions. Donor dependency also limits strategic autonomy and long-term planning. Reducing this will require diversified sponsorship models involving academic centres, philanthropic foundations, and regional funding mechanisms. However, this diversification must be approached with caution. Civil society organizations should avoid accepting support from commercial actors whose interests are not aligned with health promotion goals, such as tobacco, alcohol, arms, and other health-harming sectors. These actors often prioritize profits over public health, and engaging with them could weaken the credibility of health promotion efforts (Thomas *et al*. 2024). The second is procedural access. Even though WHO has expanded pathways for non-state actors (e.g. Official Relations for A4HP in 2015), the global system remains structurally state-centred, which slows uptake of community-generated solutions and underutilizes local legitimacy. We need governance that shares power by design.

Civil society is uniquely placed to shorten the distance between policy and people by turning abstract goals into trusted practice. It can also mobilize youth leadership and intergenerational cooperation that will determine whether reforms are sustained throughout the following decades. These advantages align with the Shanghai Declaration’s emphasis on governance, healthy cities, and health literacy and the Geneva Charter’s blueprint for well-being societies. This will require recognizing civil society as a core component of health promotion governance across all levels, from global to local.

As a result, we need to fund civil society as a public good. This will allow civil society networks to create long-lasting outcomes and deliver on the Charter’s five action areas. Predictable financing would allow them to build staff, data systems, and partnerships. In turn, this would build continuity, trust, and credibility, enabling them to report on shared indicators and publish results on open platforms, thereby making local realities more visible. Protecting and expanding civic spaces is essential. Monitoring and reporting on civic space conditions affecting health promotion should be a shared responsibility among the WHO, other international health agencies and partners, member states, and CSOs themselves. The Official Relations framework can be used to expand civil society’s seats at the governance table, where charters are translated into budgets and regulations.

We need to institutionalize two-way policy channels by involving communities in national health promotion strategies. Ministries should include civil society and youth bodies at the design, implementation, and evaluation phases. In addition, building digital communications would expand the reach and impact of health promotion strategies. We should use the GHPH to create standing communities on health literacy, youth governance, and digital determinants. Funded initiatives should share tools and data publicly and host an annual review to diffuse effective strategies. Increased communication would allow shared learning, further propelling success.

We will know we are on course when community action is not just a title but a line item in budgets, when youth leaders sit on boards involved in policy-making, when ministries regularly test policies for health impacts and equity, and when the WHO Hub shows yearly growth in shared tools, communities of practice, and international collaborations that survive election cycles.

The trail from Ottawa through Shanghai to Geneva is a curriculum for governing in difficult times. We do not need another charter to tell us what to do. We need a new settlement that treats civil society as a core institution of public health, with the mandate, resources, and digital platforms to implement what we already know works. If we enact Ottawa’s vision by focusing on community power, protecting civic space, financing participation, and building digital infrastructures, the world will better withstand unavoidable crises and seize opportunities we cannot afford to miss. That is what health promotion was always for, and now, it is what it must become.

## Data Availability

Not applicable.
